# Supplementary material for: Microglia Influence Neurofilament Deposition in ALS iPSC-Derived Motor Neurons
Source: Genes (Basel). 2022 Jan 27;13(2):241. doi: 10.3390/genes13020241 (PMC8871895; doi:10.3390/genes13020241)
Supplement: Supplementary file 1 [file genes-13-00241-s001.zip › genes-1558214-supplementary.pdf]

Microglia Influence Neurofilament Deposition in ALS iPSC-Derived Motor Neurons - Supplemental Information

Supplemental Figure S1

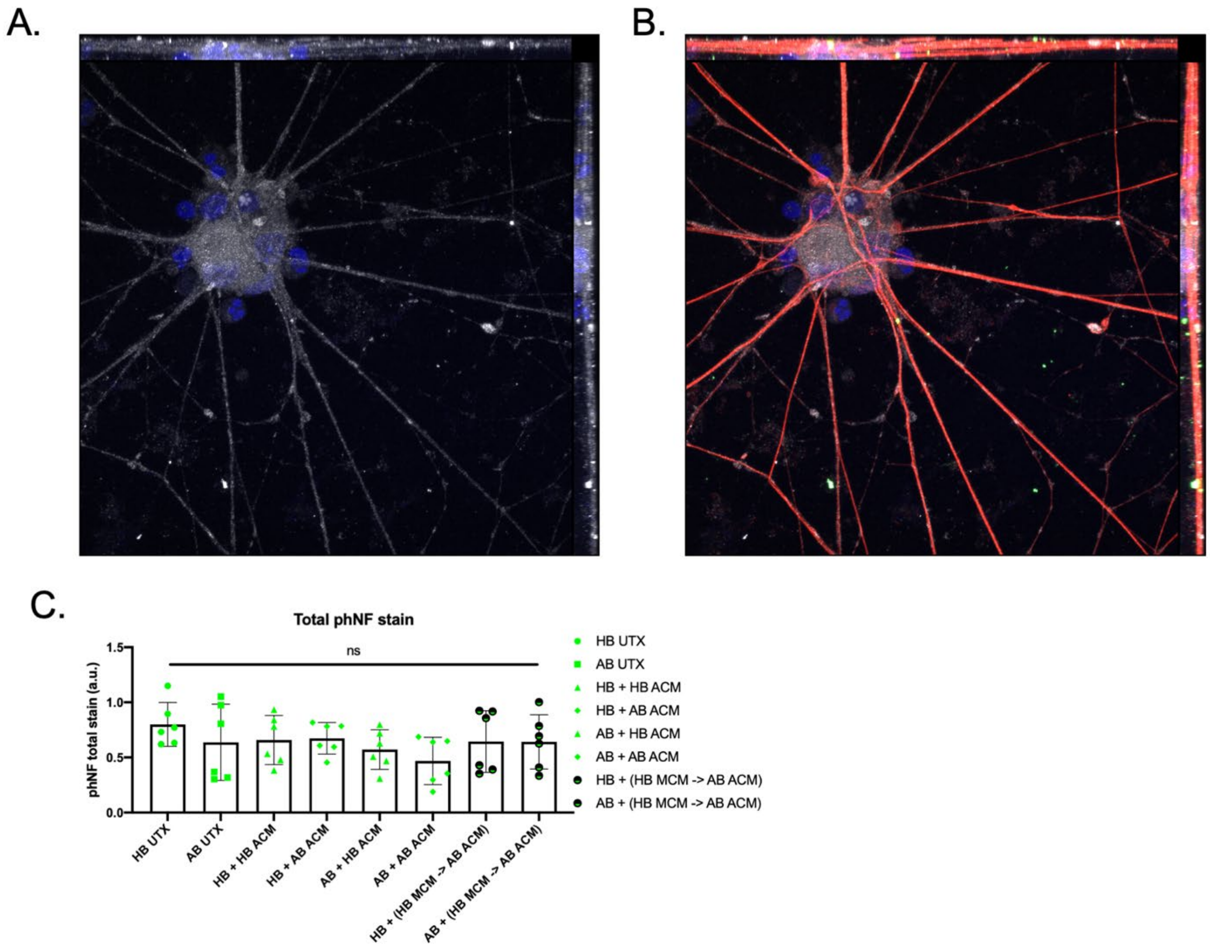

Supplemental Figure S1: Only phNF+ aggregates differ between treatment groups –not total phNF– indicative of an ALS phenotype. (**A**) All MNs quantified in ICC analyses were ChAT+ (blue = DAPI, white = ChAT). (**B**) NF200 and phNF+ aggregates colocalized with ChAT staining (blue = DAPI, white = ChAT, red = NF200, green = phNF, 63x objective). (**C**) Quantified ICC of total phNF shows no differences between HB and AB MNs, and no significant changes between treatment groups [1-way ANOVA, ns].

Supplemental Figure S2

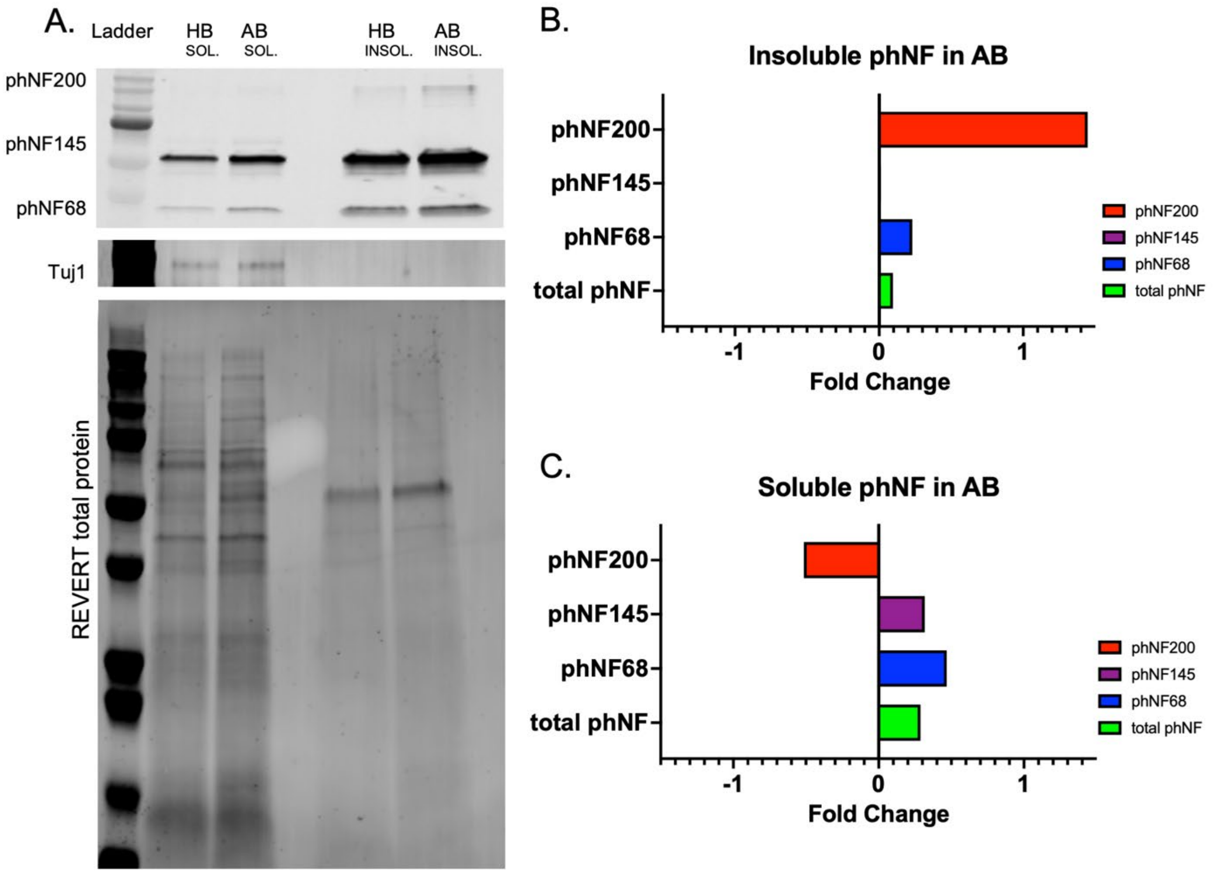

Supplemental Figure S2: Soluble and insoluble fractionation of HB and AB MN pellets. HB and AB MN pellets were separated into soluble and insoluble fractions and assessed via Western blot for phosphorylated NFs. (**A**) Blot was normalized to REVERT total protein stain and quantified for total phosphorylated NFs as well as individually analyzed for phosphorylated NF200, NF145, and NF68. Tuj1 was included to ensure proper fractionation (should not appear in insoluble fractions). Expression levels for these proteins in AB sample were normalized to HB to calculate fold change in insoluble (**B**) and soluble (**C**) fractions. Notably, phNF200 is found increased in insoluble fraction of AB compared to HB and decreased in soluble fraction. Subtle increases in total phNF in both insoluble and soluble fractions.

Supplemental Figure S3

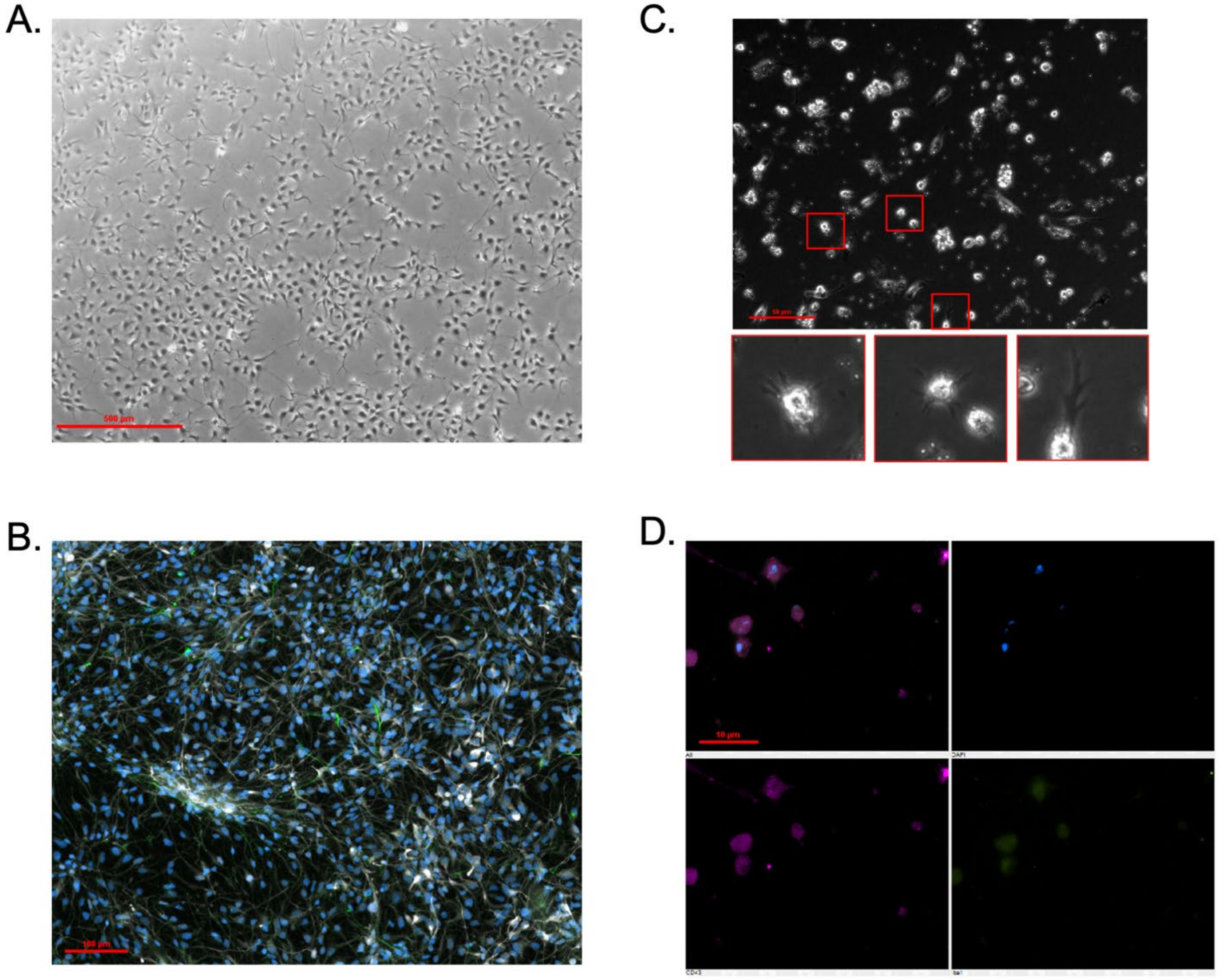

Supplemental Figure S3: Representative images of astrocytes and microglia differentiated from iPSCs. (**A**) Brightfield image of AB astrocytes p.3 (4x objective). (**B**) ICC for canonical astrocyte markers GFAP (green) and S100B (white) in AB astrocytes after p.4 (10x objective, blue = DAPI). (**C**) Brightfield image of iPSC-derived microglia on day 30 of differentiation (20x objective, red boxes show zoomed images). (**D**) ICC for canonical microglia markers Iba1 (green) and CD43 (purple) in iPSC-derived microglia (60x objective, top left panel shows merged image, blue = DAPI).

Supplemental Figure S4

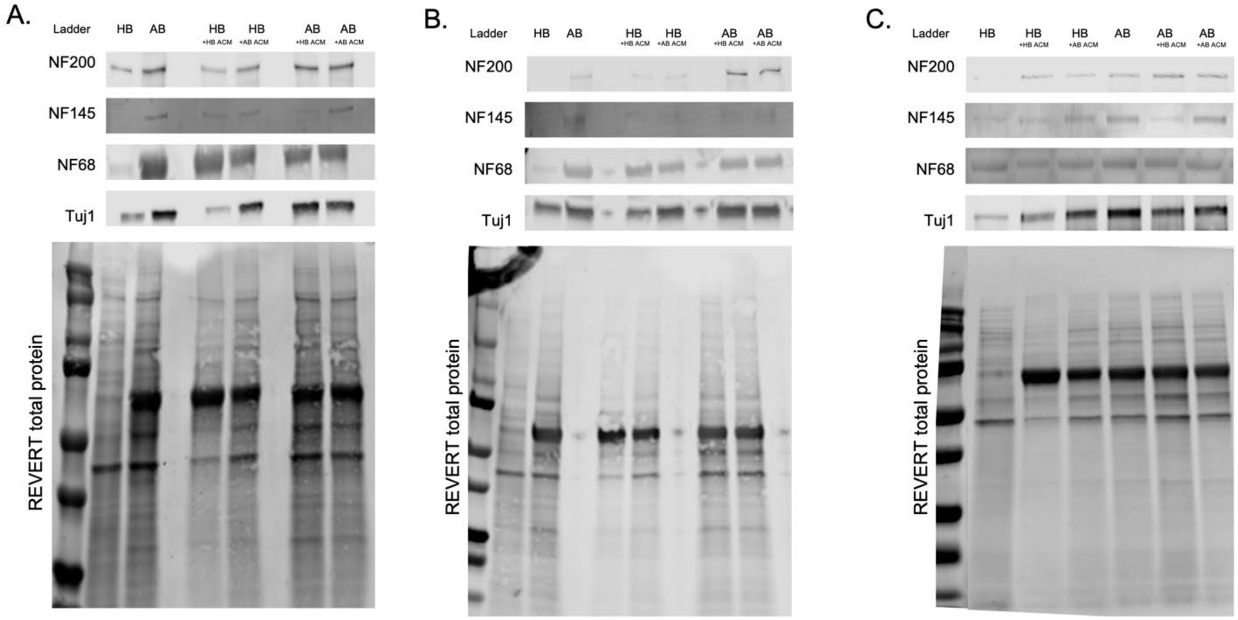

Supplemental Figure S4: Full Western blots showing untreated and treated MN samples to allow for direct comparison. Blot 1 (**A**), blot 2 (**B**), and blot 3 (**C**) used for quantification in Figures 2 and 4. Protein bands for NF200, NF145, and NF68 were normalized to REVERT total protein stain for each sample. For each experiment, untreated samples were run simultaneously with treated samples to allow for direct comparisons (quantifications in Figure 4). Tuj1 was also included as a housekeeping gene, but not used for quantification. Note that replicate 3 lanes (**C**) are in a different loading order than replicates 1 (**A**) and 2 (**B**).
